# An anoikis-related gene signature predicts prognosis and immunotherapy response, and identifies CCAR2 as a therapeutic target in triple-negative breast cancer

**DOI:** 10.3389/fimmu.2026.1808490

**Published:** 2026-05-20

**Authors:** Yan Chen, Xingwang Lu, Luying Zhang, Yunkang Li, Huilin Chen, Liang Luo, Yun Pan, Bo Gao

**Affiliations:** 1 College of Clinical Medicine, Dali University, Dali, Yunnan, China; 2 College of Basic Medicine, Dali University, Dali, Yunnan, China; 3 Department of Pathology, The First Affiliated Hospital of Dali University, Dali, Yunnan, China

**Keywords:** anoikis, CCAR2, immunotherapy, prognosis, triple-negative breast cancer

## Abstract

**Background:**

Triple-negative breast cancer (TNBC) has poor prognosis, largely due to its high rates of metastasis and recurrence. Anoikis plays a pivotal role in tumor metastasis; however, its role in TNBC remains elusive.

**Methods:**

Cox regression analysis was performed on anoikis-related genes (ARGs) to develop a prognostic signature. A clinical nomogram was developed based on the prognostic signatures. Associations of the signature with the immunogenomic landscape and response to targeted therapy and immunotherapy were assessed. Key prognostic genes were identified using random survival forest analysis. To evaluate Cell cycle and apoptosis regulator 2 (CCAR2) expression and its independent prognostic role, we performed RT-qPCR, immunohistochemistry, Kaplan-Meier analysis, and multivariate Cox regression analysis. *In vitro* functional experiments were conducted to assess the effect of CCAR2 on anoikis, migration, invasion, and stemness. Bioinformatics analyses were utilized to evaluate the association between CCAR2 expression and the immunological landscape and immunotherapy response.

**Results:**

A six-gene prognostic signature based on ARGs was constructed, stratifying patients into high- and low-risk groups with a significant difference in overall survival. The nomogram achieved good calibration and strong discriminatory accuracy. Additionally, the signature showed significant correlations with the immunogenomic landscape and predicted the response to both targeted therapy and immunotherapy. Random survival forest analysis identified CCAR2 as a key gene. In TNBC, upregulation of CCAR2 independently predicted a higher histological grade, metastasis, and poor overall survival. Its knockdown inhibits cell migration and invasion, suppresses stemness, and induces anoikis. Furthermore, CCAR2 is associated with an immunosuppressive tumor microenvironment in basal-like TNBC, fostering an immune-evasive phenotype that correlates with a poorer predicted response to immunotherapy.

**Conclusion:**

We constructed an ARG-based prognostic signature for TNBC and identified CCAR2 as a key metastatic driver that confers anoikis resistance, immune evasion, and stemness. These findings may advance the prognostic stratification of TNBC and could highlight CCAR2 as a potential therapeutic target for TNBC.

## 1 Introduction

Triple-negative breast cancer (TNBC) is defined as the absence of estrogen receptor (ER), progesterone receptor (PR), and human epidermal growth factor receptor 2 (HER2) expression (1, 2). Its poor prognosis stems primarily from the absence of therapeutic targets (3). The 5-year overall survival (OS) rate of patients with metastatic TNBC is 37.7%, with a median survival of approximately 3 years (4, 5). Ultimately, it is critical to delineate the molecular mechanisms driving metastasis and develop associated prognostic biomarkers.

Anoikis is defined as programmed cell death induced by detachment from the extracellular matrix (6). The ability of cancer cells to evade anoikis enables anchorage-independent survival, facilitating dissemination and distant colonization (7, 8). Crucially, acquiring anoikis resistance is not merely a survival mechanism, but is intrinsically linked to both immune evasion and stemness (9, 10). The synergistic interaction between these three factors significantly enhances the survival and metastatic competence of cancer cells (11–13). Therefore, a comprehensive understanding of anoikis and its interconnected mechanisms is fundamental to elucidating TNBC pathogenesis. Next-generation sequencing (NGS) has become pivotal in cancer research, generating vast amounts of omics data (14). Bioinformatics approaches are employed to analyze these data, reveal gene functions and their association with disease prognosis, and identify potential therapeutic targets (15, 16). Prognostic models based on anoikis-related genes (ARGs) have been established for various cancers, including lung adenocarcinoma (17, 18), gastric cancer (19, 20), hepatocellular carcinoma (21, 22) and colorectal cancer (23). Although studies have constructed prognostic signatures based on ARGs in breast cancer, the identification of their key genes remains unreported, and there is a lack of functional experimental validation for these prognostic genes (24–26). As a highly aggressive breast cancer subtype with poor prognosis, anoikis-related key genes may differ in TNBC.

Cell cycle and apoptosis regulator 2 (CCAR2) were cloned from the chromosomal region 8p21, which harbors a homozygous deletion in breast cancer cells (27). CCAR2 is a key regulator of diverse cellular processes, including apoptosis, metabolism, and tumorigenesis (28). However, the functional role of CCAR2 in tumorigenesis remains to be elucidated. Although CCAR2 shows increased expression in colorectal cancer (29) and hepatocellular carcinoma (30), its expression is reduced in pancreatic neoplastic lesions (31), laryngeal carcinomas, and hypopharyngeal carcinomas (32). Nevertheless, the role of CCAR2 in TNBC remains unclear.

In this study, we constructed and validated an ARG-based prognostic signature for TNBC and developed a clinical nomogram to predict survival rates. We further explored the associations of this signature with the immunogenomic landscape and responses to targeted therapy or immunotherapy and identified CCAR2 as a key prognostic gene through random survival forest analysis. Subsequently, functional studies and bioinformatic analyses revealed that it regulates anoikis resistance, stemness, and immune evasion, thereby elucidating the role of ARGs in TNBC.

## 2 Materials and methods

### 2.1 Identification of prognostic ARGs and functional enrichment analysis

We used the GeneCards database (https://www.genecards.org/) to identify ARGs. Prognostic genes for TNBC were selected by applying univariate Cox regression to genes pre-filtered using a relevance score >1. The biological functions of these prognosis-related ARGs were subsequently investigated through Gene Ontology (GO) and Kyoto Encyclopedia of Genes and Genomes (KEGG) pathway enrichment analyses with the “ClusterProfiler” R package.

### 2.2 Construction and validation of a prognostic signature

Least absolute shrinkage and selection operator (LASSO) Cox regression was employed to identify feature ARGs. The risk score was calculated as the expression level of the signature genes weighted by their regression coefficients. Following the random 4:1 split into training and testing sets, the patients were stratified into high- and low-risk groups using the median risk score derived from the training set. We used the “survival” R package to perform Kaplan-Meier analysis (KM) and the “pROC” package to generate time-dependent receiver operating characteristic (ROC) curves. Finally, we performed external validation using the independent GSE58812 cohort.

### 2.3 Construction and validation of a nomogram

We used the “rms” R package to construct a prognostic nomogram. Next, we evaluated the predictive accuracy of the nomogram using calibration curves that compared the predicted and observed outcomes. Additionally, we conducted Decision Curve Analysis (DCA) to assess the clinical net benefit. ROC curves were generated using the “pROC” package to analyze its discriminative ability.

### 2.4 Random survival forest analysis

The “Random Forest SRC” R package was employed to rank the prognostic genes based on their relative importance using a threshold of >0.2. The ranked gene importance was visualized using the” forest plot R package. Using the “survival” R package, we performed and visualized survival analysis for the six feature genes in TNBC to identify key prognostic genes.

### 2.5 Immunohistochemistry staining

The 54 TNBC specimens and 22 matched adjacent normal tissue samples were retrospectively collected at the First Affiliated Hospital of Dali University. Following fixation in 4% paraformaldehyde, the tissues were embedded in paraffin and sectioned at 4μm. Following deparaffinization and rehydration, the sections were subjected to antigen retrieval by pressure-cooking in citrate buffer for 3 min and 40 s. They were then incubated with BSA for 30 min, followed by incubation with anti-CCAR2 antibody (1:1000, ab215852, Abcam) at 37 °C for 1 h. After incubation with the secondary antibody for 30 min at room temperature, the sections were developed using DAB staining solution for six minutes. Hematoxylin-stained sections were dehydrated and mounted using a neutral resin. Staining intensity was scored as follows: 0 (negative); 1 (light yellow); 2 (brownish yellow); and 3 (brown). The percentage of positive cells was graded as 1 (≤25%), 2 (26%-50%), 3 (51%-75%), and 4 (≥75%). The two scores were multiplied to obtain the final immunoreactivity score (IRS).

### 2.6 Cell lentiviral transduction and stable clone selection

The cells were seeded in 6-well plates at a density of 3×10^5^ cells/well. For lentiviral transduction, cells were infected with CCAR2-specific shRNA lentiviral particles (Shanghai Gene Pharma Co., Ltd.) at a multiplicity of infection (MOI) of 10 in the presence of Polybrene (5μg/mL; Shanghai GenePharma Co., Ltd.). After 48 h of infection, TNBC cell lines with stable CCAR2 knockdown were selected using puromycin (1μg/mL; Shanghai GenePharma Co., Ltd.) for 48 h. The shRNA sequences were as follows (5′-3′): CCAR2-shRNA, CCTCTGAAGCAGATTAAGTTTCTC, and NC-shRNA: TTCTCCGAACGTGTCACGT.

### 2.7 Induction of anoikis

To establish a dynamic model simulating anoikis-resistant metastasis, we first seeded 3×10^5^ transfected cells into ultra-low attachment 6-well plates and cultured them in suspension for 48 h to form spheroids. These spheroids were then transferred to standard culture plates and allowed to adhere for 24 h (33).

### 2.8 Transwell assay

We seeded 3×10^4^ cells in 200μL of serum-free medium into uncoated Transwell upper chambers. For the invasion assay, the same number of cells were seeded into inserts pre-coated with Matrigel. Cells that migrated or invaded through the membrane were fixed and stained with 4% paraformaldehyde and 0.1% crystal violet, respectively.

### 2.9 Analysis of the association of CCAR2 with immunological landscape and immunotherapy response

Given the high degree of overlap, we used the basal-like subtype from the TCGA as a surrogate for TNBC for the immune landscape analyses in the TIMER3 database (https://compbio.cn/timer3/) (34), which was used to evaluate the correlation between CCAR2 expression and immune cell infiltration (CIBERSORT algorithm), T cell subsets (ImmuCellAI algorithm), and stromal cells (CONSENSUS_TME algorithm), with partial Spearman’s correlation analysis adjusted for tumor purity to determine statistical significance. It was also applied to compare CCAR2 expression levels between wild-type and mutant subgroups of PIK3CA and DMD (Wilcoxon rank-sum test), and to analyze the association of CCAR2 with immune score and the expression of 79 ICGs (35) (partial Spearman correlation analysis adjusted for tumor purity). The Assistant for Clinical Bioinformatics (https://www.aclbi.com) was utilized to explore the relationship between CCAR2 expression and gene mutation frequencies (including insertion, deletion mutations, and single nucleotide polymorphisms) via the chi-square test and to predict the immune checkpoint inhibitor (ICI) response of high and low CCAR2 expression subgroups using the Tumor Immune Dysfunction and Exclusion (TIDE) algorithm (36), with the Wilcoxon rank-sum test comparing TIDE composite scores.

### 2.10 Co-culture assay and T cell polarization marker analysis

Transwell co-culture experiments were performed using 24-well plates with 0.3 μm pore size inserts (Servicebio, China). CCAR2−knockdown MDA-MB -468 cells and negative control cells were seeded into the upper chambers at a density of 3×10^4^ cells per well. Healthy donor peripheral blood mononuclear cells (PBMCs) obtained from a commercial source (Milecell Bio, China) were added to the lower chambers at a density of 3×10^5^ cells per well, yielding an effector−to−target ratio of 10:1. PBMCs cultured alone in the lower chambers without tumor cells served as the baseline control. After 48 hours of co−culture, PBMCs were harvested from the lower chambers, and total RNA was extracted using the RNeasy Rapid Extraction Kit (GOONIE, China). cDNA synthesis and quantitative real−time PCR (qPCR) were performed as described in the **Supplementary Materials and Methods**. The following primer sequences were used for T cell subset analysis: CD3E: forward 5’−CTCCAGTAGTAAACCAGCAGCA−3’, reverse 5’−CCTGAGGGCAAGAGTGTGTG−3’. TBX21 (QH08429S) and Foxp3 (QH40125S) primer pair were purchased from Beyotime Biotechnology (China). GAPDH primer sequences were the same as described in the **Supplementary Materials and Methods**. The relative mRNA expression levels were calculated using the 2^−ΔΔCt^ method.

### 2.11 Statistical analysis

The R software (version 4.2.0) and SPSS (version 26.0) were used to analyze the data. Data are presented as mean $\left(\bar{\text{x}}\right)$ ± standard deviation (SD). Comparisons between two groups of continuous variables were performed using t-tests, whereas categorical data were compared using the chi-square test. For multi-group comparisons, one-way ANOVA followed by Dunnett’s test was used.

## 3 Results

### 3.1 Identification and functional enrichment of anoikis-related prognostic genes in TNBC

**Figure 1:**
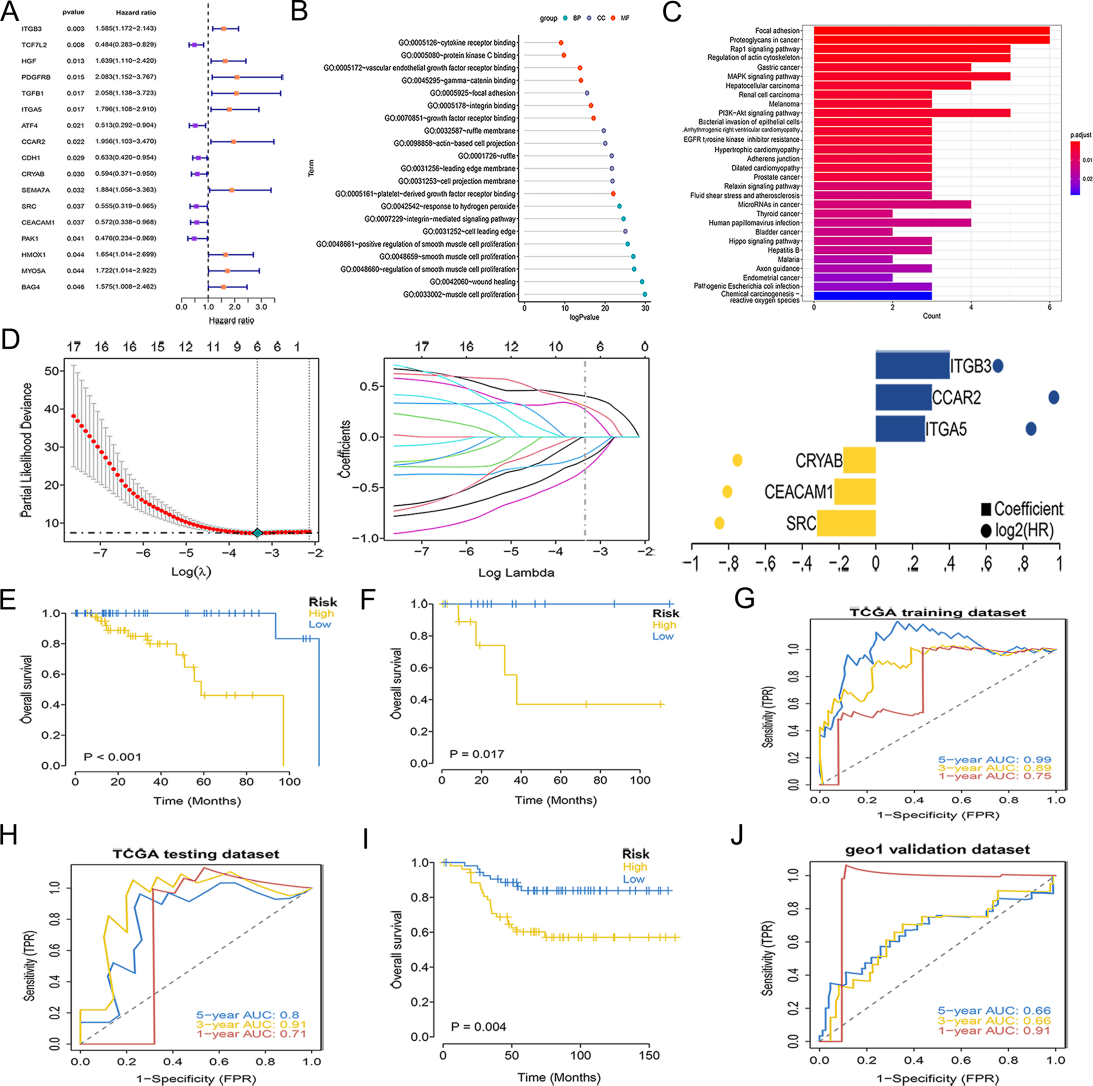
Construction and validation of the ARG signature in TNBC. **(A)** Univariate Cox regression of 17 prognostic ARGs in TNBC. **(B)** GO enrichment analysis. **(C)** KEGG enrichment analysis. **(D)** Selection of the six-gene signature via LASSO regression. **(E, F)** KM survival curves in TCGA training dataset **(E)** and testing dataset **(F)**. **(G, H)** ROC curves in TCGA training dataset **(G)** and testing dataset **(H)**. **(I)** KM survival analysis in GEO validation dataset. **(J)** ROC curve in GEO validation dataset.

We obtained 914 ARGs from the GeneCards database. After screening with a relevance score >1, 392 candidate genes were analyzed by univariate Cox regression, revealing 17 genes with significant prognostic relevance in TNBC, including ITGB3, TCF7L2, HGF, PDGFRB, TGFB1, ITGA5, ATF4, CCAR2, CDH1, CRYAB, SEMA7A, SRC, CEACAM1, PAK1, HMOX1, MYO5A, and BAG4 (**Figure 1A**). GO analysis revealed significant enrichment in all three categories (**Figure 1B**). Biological process (BP), enrichment was observed in muscle cell proliferation, smooth muscle cell proliferation, and regulation of smooth muscle cell proliferation. For cellular components (CC), enrichment was observed in focal adhesions and ruffle membranes. For molecular functions (MF), the primary enrichments were protein kinase C binding, growth factor receptor binding, and integrin binding. KEGG pathway analysis indicated significant enrichment in the focal adhesion and PI3K-AKT signaling pathways (**Figure 1C**).

### 3.2 Construction and validation of a novel prognostic ARG signature in TNBC

LASSO regression identified six feature genes: proto-oncogene C-Src (SRC), carcinoembryonic antigen-related cell adhesion molecule 1 (CEACAM1), crystallin alpha B (CRYAB), integrin subunit alpha 5 (ITGA5), CCAR2, and integrin subunit beta 3 (ITGB3). The risk score was calculated using the following formula: (SRC × -0.3197) + (CEACAM1 × -0.2254) + (CRYAB × -0.1768) + (ITGA5 × 0.2683) + (CCAR2 × 0.3055) + (ITGB3 × 0.4026) (**Figure 1D**). KM analysis revealed a significantly worse OS for high-risk patients in both the training and testing datasets (**Figures 1E, F**). The ROC curves further confirmed the strong discriminatory power of the signature, demonstrating its high predictive accuracy for both the internal datasets (**Figures 1G, H**). Validation in an independent external GEO cohort GSE58812 confirmed the generalizability of the signature, as the high-risk group consistently showed markedly inferior survival rates (**Figure 1I**). ROC analysis demonstrated that the model achieved AUCs of 0.91, 0.66, and 0.66 for predicting 1-, 3-, and 5-year OS, respectively (**Figure 1J**).

### 3.3 Independent validation and clinical association of the prognostic ARG signature in TNBC

**Figure 2:**
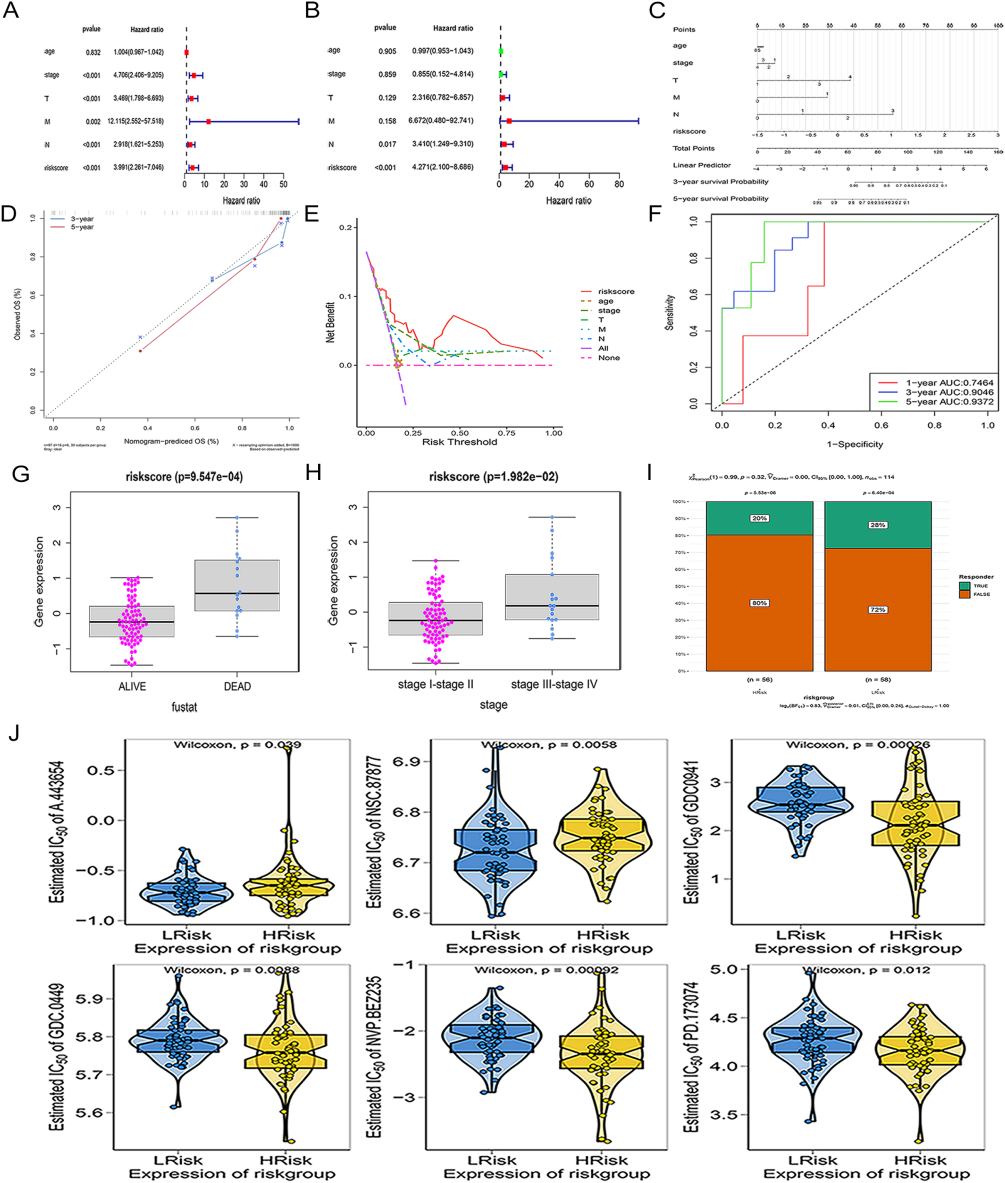
Independent validation and clinical association of the prognostic ARG signature in TNBC. **(A)** Univariate Cox regression analysis. **(B)** Multivariate Cox regression analysis. **(C)** Nomogram for predicting 3- and 5-year OS. **(D)** Calibration curve. **(E)** DCA analysis. **(F)** ROC curves. Box plots showing the distribution of risk scores according to survival status **(G)** and clinical stage **(H)**. **(I)** Association between risk groups and immunotherapy response. **(J)** The high- and low-risk groups show differential sensitivity to six chemical compounds from the GDSC database.

After initial univariate screening (**Figure 2A**), multivariate Cox analysis confirmed that the risk score and N stage were independent prognostic factors (**Figure 2B**). The prognostic nomogram (**Figure 2C**) demonstrated excellent calibration, with predicted outcomes closely aligned with actual observations (**Figure 2D**). DCA confirmed that incorporating the risk score into clinical decisions yielded a superior net benefit compared to the alternatives (**Figure 2E**). ROC analysis showed AUC values of 0.746, 0.905, and 0.937 for predicting the 1-, 3-, and 5-year OS, respectively (**Figure 2F**). Analysis of clinical correlations revealed that deceased patients had significantly higher risk scores than those of surviving patients (**Figure 2G**), and patients with stage III-IV had higher scores than those with stage I-II (**Figure 2H**). The high-risk group showed a trend toward a higher predicted non-response rate based on the TIDE algorithm, suggesting a potentially poorer response to immunotherapy that warrants further investigation (**Figure 2I**). Furthermore, the risk score was significantly predictive of sensitivity to several targeted agents, including a pan-Akt inhibitor (A-443654) and selective phosphatidylinositol 3-kinase (PI3K) inhibitor (GDC-0941) (**Figure 2J**).

### 3.4 The ARG signature is associated with the immunogenomic landscape and oncogenic pathways

**Figure 3:**
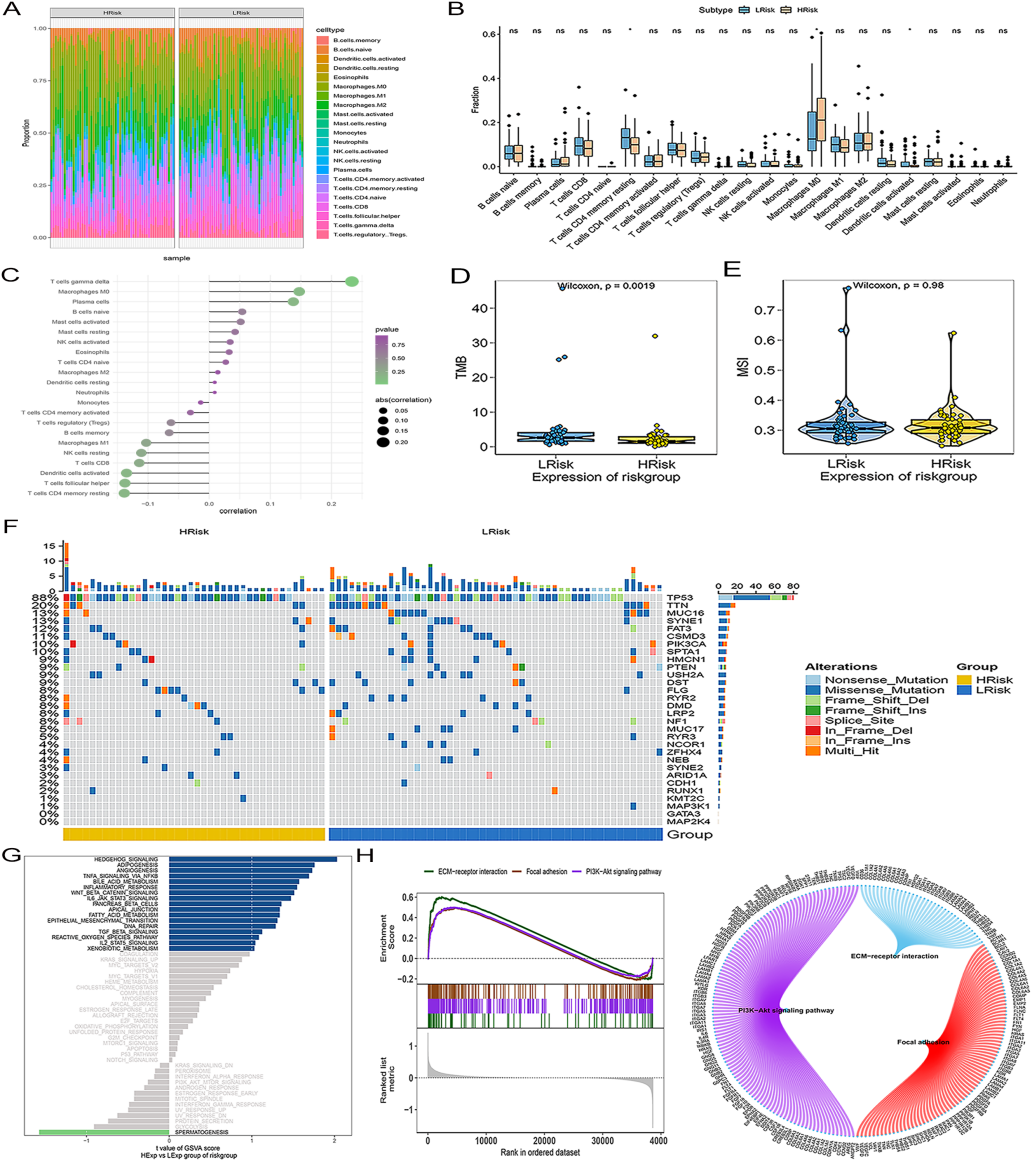
The ARG signature is associated with the immunogenomic landscape and oncogenic pathways. **(A)** CIBERSORT analysis. **(B)** Differences in immune cell abundance. **(C)** Correlation analysis between the ARGs risk score and immune cell infiltration levels. **(D)** TMB analysis. **(E)** MSI scores. **(F)** Waterfall plot depicting the gene mutation landscape. **(G)** GSVA analysis. **(H)** GSEA analysis.

Given the clinical importance of immunotherapy in TNBC, we analyzed the correlation between risk scores and established immunotherapy-related biomarkers. The distribution of immune cell types in the high- and low-risk groups is shown in **Figure 3A**. The high-risk group was characterized by a significantly lower abundance of resting CD4 memory T cells and a markedly higher abundance of M0 macrophages than the low-risk group (**Figure 3B**). We found that the risk score was positively correlated with gamma delta T cells and M0 macrophage infiltration but negatively correlated with resting CD4 memory and follicular helper T cells (**Figure 3C**). In the high-risk group, a lower TMB was observed (**Figure 3D**), and reduced mutation rates in TP53 and TTN (**Figure 3F**). Additionally, no significant differences in MSI status were observed between the risk groups (**Figure 3E**). GSVA and GSEA were performed to define associated pathways. GSVA indicated differential activity of the Hedgehog signaling, adipogenesis, and angiogenesis pathways (**Figure 3G**). Furthermore, GSEA revealed significant enrichment in pathways related to ECM-receptor interactions, focal adhesion, and PI3K-Akt pathways (**Figure 3H**).

### 3.5 Identification and characterization of key genes in TNBC

**Figure 4:**
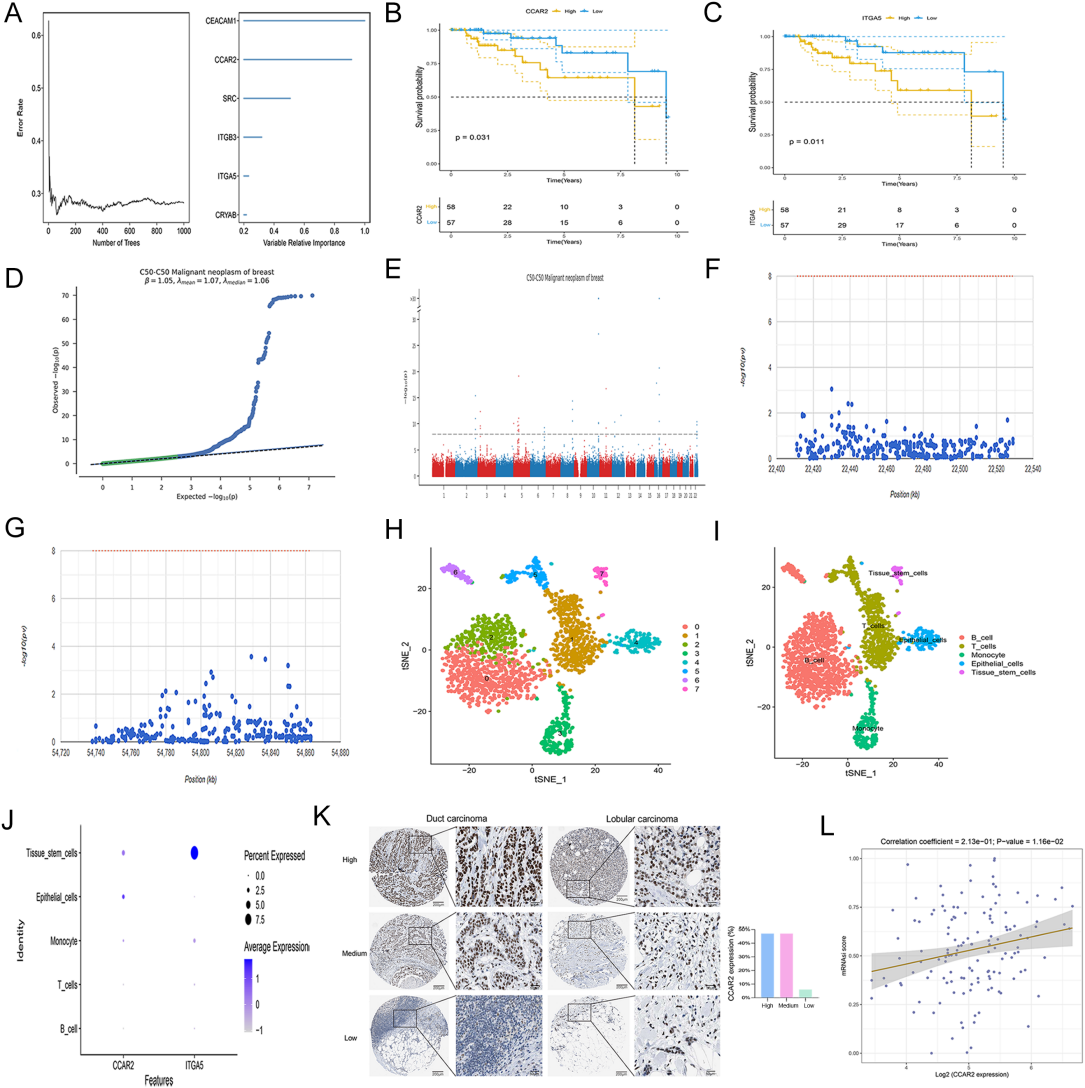
Identification and characterization of key genes in TNBC. **(A)** Random survival forest analysis. **(B, C)** KM survival analysis based on CCAR2 **(B)** and ITGA5 **(C)** expression levels. **(D)** Quantile-quantile plot. **(E)** Manhattan plot. **(F)** CCAR2 is located in the pathogenic region of chromosome 8. **(G)** ITGA5 is located in the pathogenic region of chromosome 12. **(H)** Cell clustering identified eight subpopulations. **(I)** Annotation of the eight cellular clusters into five cell types. **(J)** Expression of CCAR2 and ITGA5 in different cell types. **(K)** Representative IHC images of CCAR2 expression in breast cancer from the HPA database. **(L)** Correlation between CCAR2 expression and stemness in TNBC.

The six signature genes were ranked in the following order by importance using random survival forest analysis: CEACAM1, CCAR2, SRC, ITGB3, ITGA5, and CRYAB (**Figure 4A**). High expression of CCAR2 and ITGA5 was associated with poorer prognosis (**Figures 4B, C**). Next, the pathogenic regions of these two key genes were identified (**Figures 4D, E**). CCAR2 was mapped to the pathogenic region on chromosome 8 (**Figure 4F**), and ITGA5 in the region on chromosome 12 (**Figure 4G**). Single-cell analysis of the data from GSE180286 revealed eight distinct cell subpopulations (**Figure 4H**). These subpopulations were annotated as the following five cell types: B cells, T cells, monocytes, epithelial cells, and tissue stem cells (**Figure 4I**). Among these, tissue stem cells exhibited higher expression of CCAR2 and ITGA5 than the other tissues. (**Figure 4J**). Based on prioritized ranking, CCAR2 protein expression patterns in breast cancer were profiled using the HPA database. The results demonstrated that CCAR2 was localized in the nucleus (**Figure 4K**). Among the 34 breast cancer cases, moderate or high CCAR2 expressions were observed in 32 cases. Finally, CCAR2 expression showed a significant positive correlation with the stemness score in TNBC (**Figure 4L**).

### 3.6 CCAR2 is an independent prognostic biomarker in TNBC

**Figure 5:**
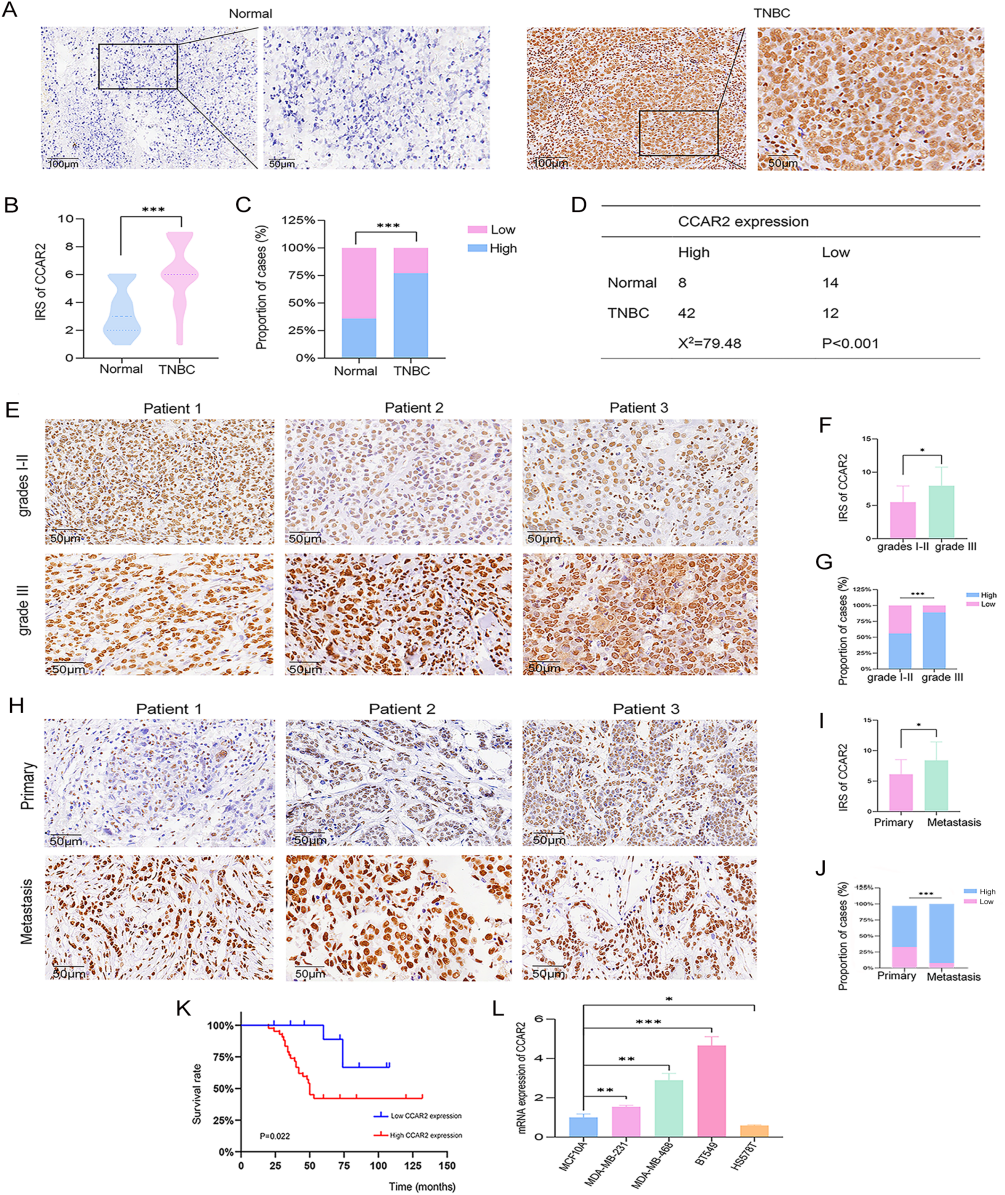
CCAR2 is an independent prognostic biomarker for TNBC. **(A)** Representative IHC images of CCAR2 expression in adjacent normal and TNBC tissue. **(B)** CCAR2 protein expression was elevated in TNBC compared to adjacent normal tissue. **(C, D)** CCAR2 expression was significantly higher in TNBC than in adjacent normal tissues. **(E)** Representative IHC images of CCAR2 expression in grade I-II and III TNBC. **(F, G)** Association between CCAR2 protein expression and histological grade in TNBC. **(H)** Representative IHC images of CCAR2 expression in primary and metastatic cancers. **(I, J)** Comparative analysis of CCAR2 expression in primary and metastatic TNBC. **(K)** High CCAR2 protein expression correlates with poor prognosis in TNBC. **(L)** RT-qPCR analysis of CCAR2 mRNA expression levels in TNBC cell lines and the normal mammary epithelial cell line MCF10A. *P < 0.05, **P < 0.01, ***P < 0.001.

CCAR2 expression was assessed by IHC in 54 TNBC specimens and 22 adjacent normal tissue specimens. We observed that CCAR2 was markedly overexpressed in TNBC tissues compared to normal tissues (**Figures 5A, B**) and was highly expressed in most TNBC cases (**Figures 5C, D**). High CCAR2 expression correlated with poor prognostic features, including advanced grade and metastasis (**Table 1**). Representative images and quantitative analysis revealed that CCAR2 expression was higher in grade III tumors than in grades I-II tumors (**Figures 5E–G**). Similarly, CCAR2 expression was higher in metastatic lesions than that in primary tumors (**Figures 5H–J**). Advanced histological grade, metastasis, and high CCAR2 expression were independent risk factors for poor TNBC prognosis (**Table 2**). Furthermore, KM analysis confirmed that high CCAR2 expression was associated with significantly worse OS (**Figure 5K**). Finally, RT-qPCR confirmed that CCAR2 expression was elevated in TNBC cell lines, including MDA-MB-468, MDA-MB-231, and BT-549, compared with that in the normal mammary epithelial cell line MCF10A (**Figure 5L**).

**Table 1:** The correlation between CCAR2 expression and clinicopathological features of 54 TNBC patients.

Characteristics	No. of patients	CCAR2 expression		p-value
		Low (IRS <6)	High (IRS ≥6)	
Age (n, %)				0.244
≤50 years	28	8(28.6%)	20(71.4%)	–
>50 years	26	4(15.4%)	22(84.6%)	–
Tumor size (n, %)				0.184
<3 cm	18	6(33.3%)	12(66.7%)	–
≥3 cm	36	6(16.7%)	30(83.3%)	–
Lymph Node Metastasis (n, %)				0.462
Positive	31	8(25.8%)	23(74.2%)	–
Negative	23	4(17.4%)	19(82.6%)	–
Pathological type (n, %)				0.734
IDC	35	7(20.0%)	28(80.0%)	–
All others	19	5(26.3%)	14(73.7%)	–
Histological grade (n, %)				0.012
I/II	18	8(44.4%)	10(55.6%)	–
III	36	4(11.1%)	32(88.9%)	–
Ki-67 (n, %)				0.999
<14%	4	1(25.0%)	3(75.0%)	–
≥14%	50	11(22.0%)	39(78.0%)	–
Tumor site (n, %)				0.032
Primary	30	10(33.3%)	20(66.7%)	–
Metastasis	24	2(8.3%)	22(91.7%)	–

**Table 2:** Univariate and multivariate Cox Regression analysis of prognostic factors of the 54 TNBC patients.

Characteristics	Univariate analysis		Multivariate analysis	
	HR (95% CI)	p-value	HR (95% CI)	p-value
Age				
≤50 years	Reference	–	Reference	–
>50 years	0.674(0.278-4.983)	0.983	0.217(0.193-3.341)	0.698
Tumor size (cm)				
<3 cm	Reference	–	Reference	–
≥3 cm	0.591(0.145-2.37)	0.794	0.876(0.591-1.978)	1.457
Lymph Node Metastasis				
Positive	Reference	–	Reference	–
Negative	0.334(0.273-3.156)	0.346	0.259(0.098-2.935)	0.598
Pathological type				
IDC	Reference	–	Reference	–
All others	0.142(0.078-1.283)	0.145	0.351(0.143-1.957)	0.276
Histological grade				
I/II	Reference	–	Reference	–
III	1.518(0.954-5.231)	0.017	1.967(0.278-4.945)	0.013
Ki-67(n, %)				
<14%	Reference	–	Reference	–
≥14%	1.128(0.895-3.451)	1.751	0.934(0.278-2.839)	1.256
Tumor site				
Primary	Reference	–	Reference	–
Metastasis	1.294(0.468-1.576)	0.026	1.024(0.278-1.967)	0.034
CCAR2 expression				
Low (IRS score<6)	Reference	–	Reference	–
High (IRS score≥6)	1.745(0.957-2.539)	0.001	1.354(0.899-1.979)	0.001

### 3.7 CCAR2 knockdown induced anoikis of TNBC cells

**Figure 6:**
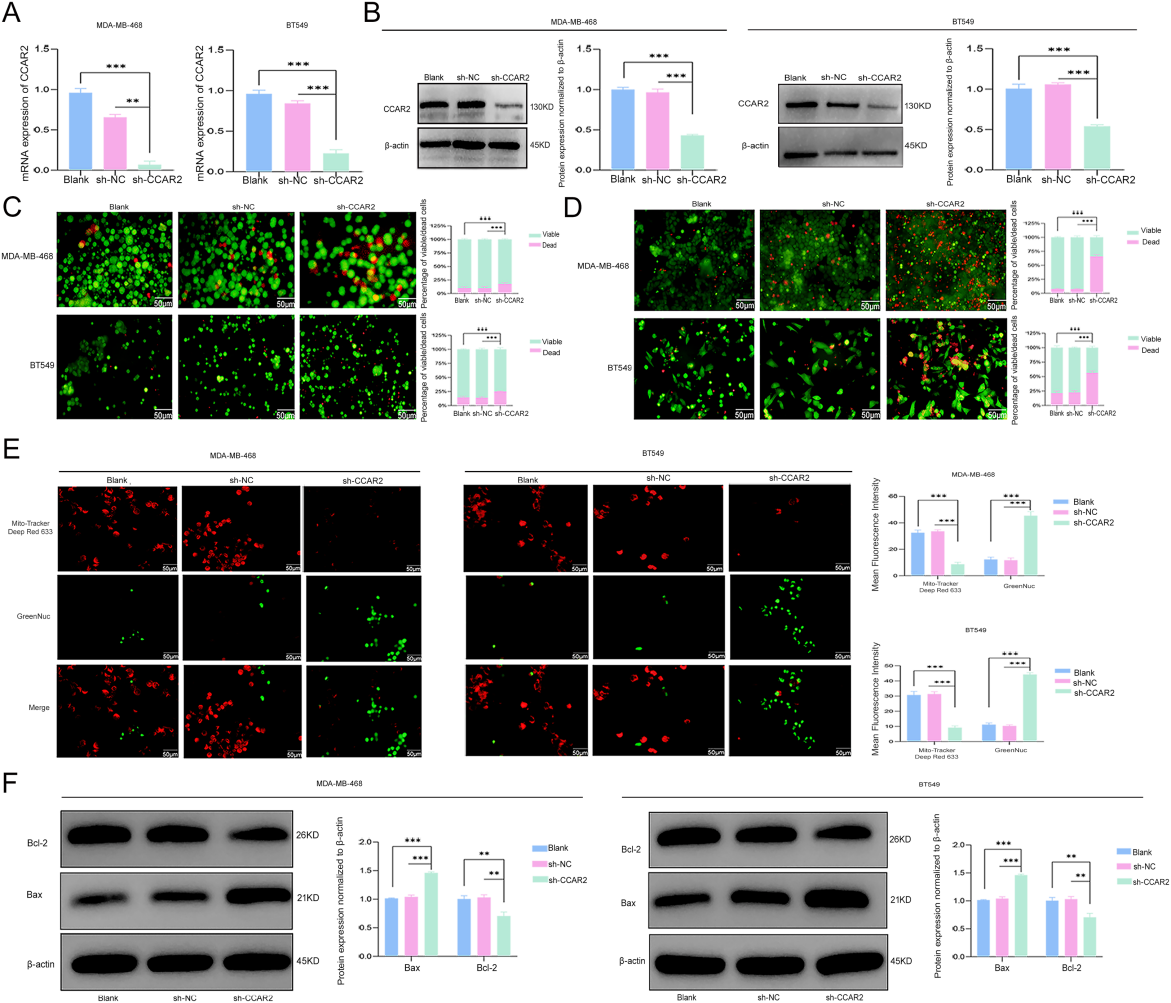
CCAR2 knockdown induced anoikis in TNBC cells. CCAR2 knockdown efficiency in TNBC cells was assessed using RT-qPCR **(A)** and western blotting **(B)**. **(C)** Assessment of viability and apoptosis in TNBC cells upon CCAR2 knockdown after detachment culture by Calcein-AM/EthD-1 staining. **(D)** Assessment of viability and apoptosis in TNBC cells upon CCAR2 knockdown after attachment culture by Calcein-AM/EthD-1 staining. **(E)** Assessment of mitochondrial membrane potential and Caspase-3 activity in TNBC cells following CCAR2 knockdown after detachment culture. **(F)** Protein levels of Bcl-2 and Bax were detected by western blotting in cells with CCAR2 knockdown after detachment culture. **P < 0.01, ***P < 0.001.

Cells with stable CCAR2 knockdown were generated for further analysis. Following the confirmation of efficient CCAR2 knockdown (**Figures 6A, B**), the cells were subjected to detachment culture for 48 h, followed by reattachment culture for 24 h. Cell viability and death were assessed using calcein-AM/ethidium homodimer I (EthD-I) double staining. CCAR2 knockdown suppressed cell viability and enhanced apoptosis compared to control cells (**Figures 6C, D**). In suspension, CCAR2 knockdown triggered loss of mitochondrial membrane potential (ΔΨm) loss and Caspase−3 activation (**Figure 6E**). Western blot analysis further demonstrated that CCAR2 knockdown shifted the balance between apoptotic regulators, reduced Bcl-2 levels, and increased Bax levels (**Figure 6F**).

### 3.8 CCAR2 knockdown inhibited migration, invasion, and stemness of TNBC cells

**Figure 7:**
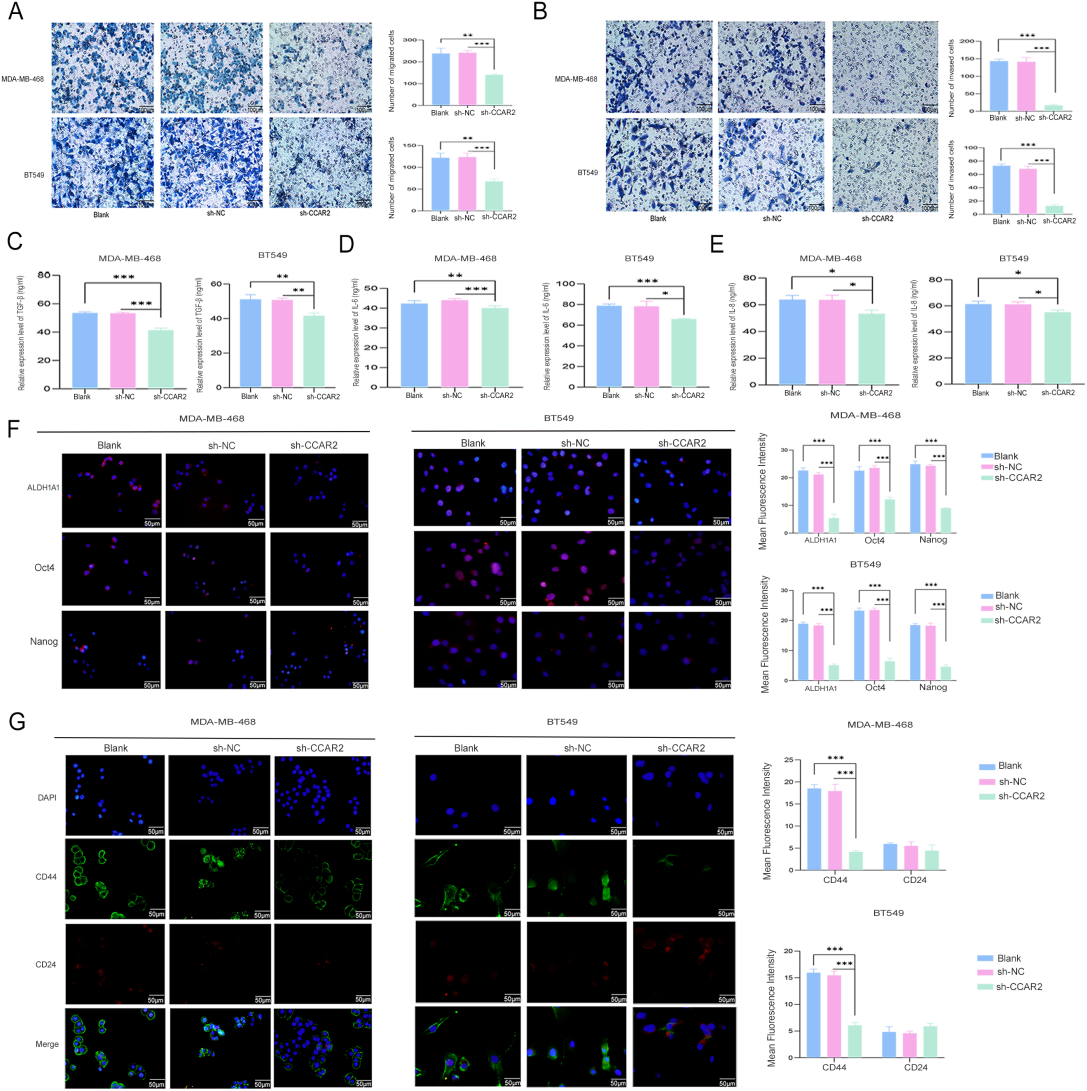
CCAR2 knockdown inhibited migration, invasion, and stemness of TNBC cells. Transwell assays assessed the migration **(A)** and invasion **(B)** abilities of TNBC cells with CCAR2 knockdown after detachment culture. Secretion of TGF-β **(C)**, IL-6 **(D)**, and IL-8 **(E)** from cells with CCAR2 knockdown after detachment culture, as determined by ELISA. **(F)** Expression of ALDH1A1, Oct4, and Nanog was analyzed by IF staining in cells with CCAR2 knockdown after detachment culture. **(G)** Expression of CD44 and CD24 was analyzed by IF staining in cells with CCAR2 knockdown after detachment culture. *P < 0.05, **P < 0.01, ***P < 0.001.

Following detachment culture, we assessed the metastatic potential using Transwell assays. The results revealed that CCAR2 knockdown significantly reduced both cell migration and invasion (**Figures 7A, B**). The role of CCAR2 in regulating stemness was investigated by assessing cancer stem cell (CSC)-related cytokine secretion via ELISA and by measuring stemness marker expression using immunofluorescence. A significant reduction in the secretion of TGF-β, IL-6, and IL-8 was observed in cells with CCAR2 knockdown (**Figures 7C–E**). Furthermore, diminished fluorescence intensities of ALDH1A1, Oct4, and Nanog were observed following CCAR2 knockdown (**Figure 7F**). Consistent with this, CCAR2 knockdown reduced the number of CD44+/CD24- cells (**Figure 7G**).

### 3.9 CCAR2 expression correlates with tumor immune landscape and immunotherapy response

**Figure 8:**
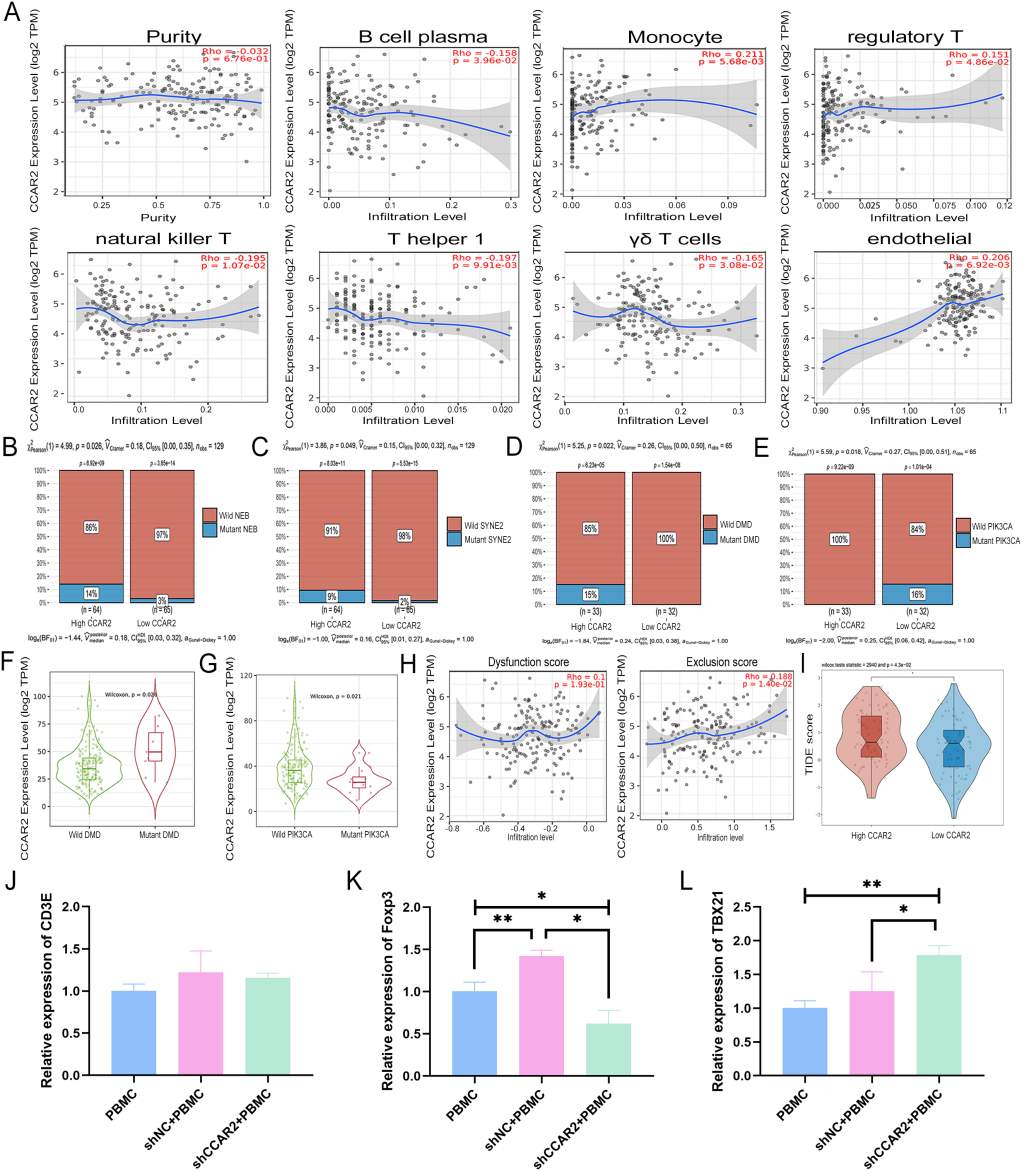
CCAR2 expression correlates with the tumor immune landscape and immunotherapy response, and affects T cell polarization. **(A)** Correlation between CCAR2 expression and infiltration levels of immune and stromal cells in the tumor microenvironment of basal-like breast cancer from the TCGA cohort. **(B-E)** Comparison of mutation frequencies of NEB **(B)**, SYNE2 **(C)**, DMD **(D)**, and PIK3CA **(E)** between high- and low-CCAR2 expression groups. **(F, G)** Differences in CCAR2 expression levels in DMD **(F)** and PIK3CA **(G)** wild-type vs. mutant subgroups. **(H)** Correlation of CCAR2 expression with tumor immune dysfunction and exclusion scores. **(I)** The TIDE algorithm was used to predict potential immune therapy response. **(J–L)** Relative expression of CD3E **(J)**, Foxp3 **(K)**, and TBX21 **(L)** in PBMCs after co−culture with CCAR2−knockdown or negative control MDA-MB-468 cells. *P < 0.05, **P < 0.01.

To systematically explore the regulatory role of CCAR2 in the tumor immune microenvironment of TNBC, we evaluated the association between CCAR2 expression and immune and stromal cell infiltration, genetic alterations, immune scores, and immunotherapy responses using multiple bioinformatics algorithms in basal-like breast cancer, which largely overlaps with TNBC and exhibits high immune cell infiltration and frequent genetic alterations. A modest but statistically significant correlation was observed between CCAR2 expression and the infiltration of several immune cell types. Specifically, CCAR2 expression was negatively correlated with the infiltration of plasma cells, natural killer T (NKT) cells, T helper 1 (Th1) cells, and γδ T cells (**Figure 8A**). In contrast, CCAR2 expression was positively correlated with that of monocytes, regulatory T (Treg) cells, and endothelial cells (**Figure 8A**). The CCAR2-high expression group had significantly higher mutation rates in NEB, SYNE2, and DMD than the CCAR2-low expression group did (**Figures 8B–D**). Conversely, the mutation rate of PIK3CA was lower in the CCAR2-high group (**Figure 8E**). Consistently, the expression level of CCAR2 was lower in the wild-type DMD subgroup than in the mutant DMD subgroup (**Figure 8F**), whereas it was higher in the wild-type PIK3CA subgroup than that in the mutant PIK3CA subgroup (**Figure 8G**). Tumor immune score analysis showed that CCAR2 expression was not significantly correlated with the dysfunction score but was positively correlated with the exclusion score (**Figure 8H**). The TIDE composite score predicted a worse response to immune checkpoint inhibitors in the CCAR2-high expression group (**Figure 8I**), which is consistent with the features of the immune landscape. Additionally, CCAR2 expression was positively correlated with 26 ICGs, including T-cell exhaust-related molecules, such as CD274 (PD-L1), PDCD1 (PD-1), LAG3, HAVCR2 (TIM-3), and CTLA4, as well as co-stimulatory molecules, such as CD276, CD40, CD86, and CD80 (**Supplementary Table 1**).

### 3.10 CCAR2 knockdown in TNBC cells modulates T cell polarization

To functionally validate the immunomodulatory role of CCAR2, we performed Transwell co−culture experiments using PBMCs and MDA-MB-468 cells, with PBMCs alone as a baseline control. After 48 hours of co−culture, PBMCs were harvested for qPCR analysis of T cell subset markers. No significant difference in CD3E expression was observed among the three groups, indicating comparable numbers of T cells in each condition (**Figure 8J**). Compared with PBMC-only control, co−culture with shNC cells significantly increased the expression of Foxp3, a lineage-defining transcription factor of Tregs, indicating that TNBC cells promote Treg differentiation. Notably, CCAR2 knockdown significantly reduced Foxp3 expression compared with shNC control (**Figure 8K**). Conversely, the expression of TBX21 (T-bet), a specific transcription factor for Th1 differentiation, was significantly increased in the shCCAR2 co−culture group compared with both PBMC-only and shNC controls, while shNC control did not differ significantly from PBMC-only group (**Figure 8L**). These results suggest that CCAR2 knockdown in TNBC cells may inhibit Treg differentiation and promote Th1 polarization.

## 4 Discussion

As a crucial barrier to tumor metastasis, anoikis is a promising therapeutic target (37). This is particularly relevant for TNBC, which is characterized by a high recurrence rate, strong metastatic potential, and short OS (38). In recent years, tumor prognostic signatures have been developed using gene sets that reflect specific biological features of the tumor (39). Therefore, we constructed an ARG-based prognostic signature for TNBC to optimize risk stratification, accurately predict prognosis, and identify novel therapeutic targets.

From an initial set of 392 ARGs, univariate Cox regression analysis identified 17 genes with significant prognostic relevance. Subsequently, a six-gene prognostic signature was derived using LASSO regression. Among these signature genes, SRC, CRYAB, ITGA5, and ITGB3 have been investigated in TNBC (40–43). SRC mediates YAP1 tyrosine phosphorylation, inducing its interaction with KLF5 to form a complex, ultimately enhancing stemness and metastasis in TNBC (40). Among the molecular subtypes of TNBC, the basal-like 2 (BL2) subtype exhibits the lowest post-chemotherapy survival rate and highest risk of metastasis. One study showed that CRYAB expression is elevated in the BL2 subtype compared to other subtypes and is associated with brain metastasis (41). ITGA5 is overexpressed in highly migratory and invasive TNBC cells and their lung metastatic foci in nude mice; importantly, the delivery of ITGA5-targeted nanoparticles can downregulate β-catenin and effectively suppress TNBC growth and metastasis (42). In TNBC, thrombomodulin negatively regulates ITGB3 expression by activating the Smad2/3 pathway, whereas elevated ITGB3 expression promotes cell proliferation and migration (43). To date, the functional mechanisms of CCAR2 and CEACAM1 in TNBC have not yet been elucidated.

This robust and reproducible six-gene signature successfully stratified TNBC patients into high- and low-risk groups, with consistent and significant differences in overall survival across all datasets. Thus, this signature not only serves as a reliable prognostic predictor but also holds promise for guiding future treatments. This potential is supported by the favorable performance of the corresponding nomogram, as evidenced by the good predictive accuracy of the calibration curve and the superior net clinical benefit of the risk score in the DCA. More importantly, a high-risk score was used to identify patients with both shorter survival and poorer response to immunotherapy. In terms of targeted therapy, high-risk tumors exhibit a divergent sensitivity profile, showing resistance to AKT inhibition, but heightened sensitivity to PI3K inhibition. Interestingly, KEGG and GSEA analyses revealed significant enrichment of the PI3K-AKT signaling pathway. Collectively, these results suggest that the risk signature can be used to stratify patients for therapy development. This aligns with the role of dysregulated PI3K-AKT signaling as a frequent oncogenic driver of TNBC (44). Accumulating evidence suggests that targeted therapy can generate immunological effects by inducing interactions between tumor and immune cells (45). Our analysis revealed that the high-risk group was associated with a significant reduction in resting CD4^+^ memory T cells, lower TMB, and lower mutation rates in TP53 and TTN. These features likely contributed to the poor response to immunotherapy observed in this group.

In this study, CCAR2 emerged as the top-ranking prognostic gene in the random survival forest analysis. However, the role of CCAR2 in TNBC tumorigenesis remains unclear. It has been reported that CCAR2 is downregulated or upregulated, and acts as a prognostic indicator of either favorable or unfavorable outcomes in various cancers, even in the same malignancy, such as breast cancer (46). Lee et al. reported high CCAR2 expression in 71% of patients with breast cancer, linking it to distant metastasis recurrence and predicting poor recurrence-free survival and OS (47). Conversely, CCAR2 deficiency promotes the proliferation and migration of breast cancer cells (48). A previous study proposed that CCAR2 acts as either a tumor suppressor or promoter, depending on TP53 mutation status (46). TP53 is mutated in 42% of tumors across cancer types (49), and these mutant proteins lose their tumor-suppressive functions and acquire oncogenic gain-of-function properties, thereby driving cancer progression (49). For instance, CCAR2 stabilizes gain-of-function p53 mutants, such as R280K, by inhibiting ubiquitination, thereby enhancing cancer cell survival and chemoresistance (50). Furthermore, the co-occurrence of high CCAR2 expression and TP53 mutations predicts poorer outcomes in gliomas (28). TP53 is deleted or mutated in approximately 84% of TNBC cases (51), suggesting that TP53 alterations are critical drivers of TNBC. Our study showed that CCAR2 is upregulated in TNBC and correlated with advanced tumor grade, metastasis, and poor survival, suggesting its oncogenic role in TNBC. However, no significant correlation was observed between CCAR2 expression and TP53 mutation status within the basal-like subset, which may be attributed to the limited sample size in this subset.

The role of CCAR2 in tumor metastasis, particularly through the regulation of anoikis, remains poorly understood. In gastric cancer, CCAR2 activates IKK-β/NF-κB signaling, thereby conferring resistance to anoikis and facilitating metastasis (52). In the present study, CCAR2 knockdown induced anoikis and impaired the migration and invasion of TNBC cells. We also observed a higher proportion of dead cells in the re−attached state than in the suspension culture state. A possible explanation for the delayed apoptosis upon reattachment is that CCAR2 knockdown not only disrupts survival signaling under suspension conditions by reducing the Bcl−2/Bax ratio, impairing mitochondrial membrane potential, and activating caspase−3, but also impairs the ability of cells to reattach and re−establish extracellular matrix connections. Under suspension conditions, some cells survive but become vulnerable, as evidenced by mitochondrial damage. The reattachment process requires rapid reorganization of adhesion plaques, activation of integrin signaling including ITGB3 and ITGA5, and restoration of mitochondrial function to deliver survival signals. Of note, both ITGB3 and ITGA5 are also genes included in our prognostic signature. CCAR2 knockdown may prevent cells from transmitting these survival signals upon re−exposure to the matrix, and the demands of re−adhesion may exceed the capacity of the cells, thereby triggering apoptosis at this stage. Future studies using integrin−specific inhibitors or rescue experiments are warranted to further elucidate the underlying molecular mechanisms.

The regulatory mechanism of anoikis is critical for epithelial cells owing to its high renewal rate. Anoikis resistance is necessary for sphere formation during the metastatic progression of cancer cells and is a well-known feature of CSCs (53). Interestingly, CSC-like cells can protect non-stem cells from anoikis and promote mammosphere formation and xenograft tumor growth (54). This bystander effect is thought to be mediated by cytokine/chemokine-containing exosomes derived from CSC-like cells, which activate the JAKs-STAT3-Bcl-xL signaling pathway in non-stem cells (54). Our data demonstrated elevated CCAR2 expression in tissue stem cells and a positive correlation with stemness scores in TNBC. One study showed that CCAR2 is required for the CSC-like properties of colon cancer cells and promotes colorectal cancer progression via the Wnt/β-catenin-MACC1 signaling axis (29). CCAR2 knockdown also potently suppressed stemness, as evidenced by the reduced expression of key markers ALDH1A1, Oct4, Nanog, and CD44.

CSCs in solid tumors exhibit unique immune-evasive properties that enable them to drive tumor regrowth (55). The basal-like subtype shows the highest stemness compared to other breast cancer subtypes, and stemness is negatively associated with immune cell infiltration in solid cancers (56). Our study revealed that high CCAR2 expression enriched Tregs while excluding effector cells such as plasma, NKT, and Th1 cells, which is associated with an immunosuppressive tumor microenvironment (TME) in basal-like breast cancer. This study further demonstrated that CCAR2 knockdown in TNBC cells significantly reduced Foxp3 expression and increased TBX21 expression in co−cultured PBMCs, supporting a role of CCAR2 in promoting Treg differentiation and suppressing Th1 polarization. The TIDE exclusion score is a computational prediction based on transcriptomic signatures of immunosuppressive cells, but this approach has been extensively validated in clinical cohorts and predicts immunotherapy response with high accuracy (36). The importance of tumor endothelial cells in the TME for promoting angiogenesis, tumor growth, and immune evasion has been increasingly recognized (57). Accumulating evidence indicates that tumor endothelial cells play a critical role in T cell exclusion by forming a physical barrier and through molecular mechanisms such as Rap1B signaling and metabolic reprogramming (58, 59). Therefore, the positive correlations of CCAR2 with both the TIDE exclusion score and endothelial cell infiltration in this study further support the immunosuppressive role of CCAR2. In addition, CCAR2 expression positively correlated with the expression of 26 ICGs, including CD 274, PD-1, LAG3, and TIM-3. These features may explain the poor response to checkpoint inhibitors in patients with high CCAR2 expression. However, we acknowledge that basal-like and TNBC are not entirely synonymous, and our findings derived from basal-like datasets should be interpreted with caution and ideally validated in TNBC cohorts.

Despite these findings, this study had several limitations. First, the predictive performance of our prognostic signature showed a decrease in AUC when validated in the external GSE58812 cohort compared to the TCGA training set. This difference is likely attributable to inherent heterogeneity between cohorts, such as differences between multi−center and single−center study designs as well as technical variations between RNA−seq and microarray platforms. Importantly, this decline in AUC should not be interpreted as model overfitting, because the signature still significantly stratified patients into high− and low−risk groups with distinct survival outcomes in the validation set. Nevertheless, we acknowledge that cross−platform validation remains a challenge in bioinformatics studies. Future prospective studies using larger multi−center cohorts with standardized detection platforms are warranted to further confirm the robustness and clinical utility of this signature. Second, the association between our prognostic signature and immunotherapy response was based on the TIDE algorithm, and the observed difference in predicted non-response rates between high- and low-risk groups was modest. Therefore, the predictive value of this signature for immunotherapy response should be validated in real-world clinical cohorts of TNBC patients receiving immune checkpoint inhibitors. Third, the immunohistochemistry validation of CCAR2 expression was based on a relatively small cohort, and larger cohort studies are needed to confirm the prognostic value of CCAR2. Fourth, our functional validation of the immunomodulatory role of CCAR2 was limited to assessing Foxp3 and TBX21 expression in PBMCs, without cytokines detection or T cell cytotoxicity assays. In addition, due to the lack of *in vivo* animal experiments in this study, the functional role of CCAR2 in an intact immune microenvironment remains to be validated. Future studies employing immunocompetent mouse models are essential to confirm the immunomodulatory role of CCAR2. Finally, the precise molecular mechanisms through which CCAR2 regulates immune evasion, anoikis resistance, and cancer stemness warrant further in−depth investigation and *in vivo* validation.

## 5 Conclusion

In summary, we established a novel and robust prognostic signature based on ARGs for TNBC, which may serve as a practical tool for the prognosis assessment and clinical management of patients with TNBC. Notably, our findings established a key role for CCAR2 in TNBC metastasis, orchestrating anoikis resistance, immune evasion, and stem cell-like properties, providing a rationale for its use in prognostic stratification and as a therapeutic target for TNBC.

## Data Availability

The original contributions presented in the study are included in the article/**Supplementary Material**. Further inquiries can be directed to the corresponding author.
