# Supplementary material for: An anoikis-related gene signature predicts prognosis and immunotherapy response, and identifies CCAR2 as a therapeutic target in triple-negative breast cancer
Source: Front Immunol. 2026 May 20;17:1808490. doi: 10.3389/fimmu.2026.1808490 (PMC13229996; doi:10.3389/fimmu.2026.1808490)
Supplement: Supplementary file 1 [file DataSheet1.docx]

Supplementary Material

**Images of Western blot.**

**Figure 7B**

CCAR2 expression in MDA-MB-468 cell：


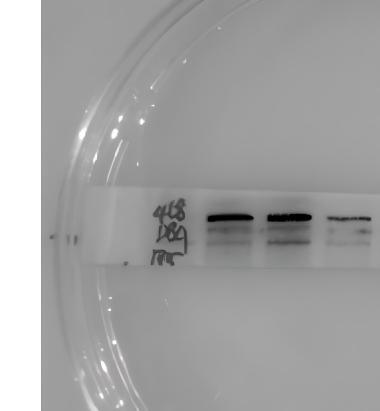


β-actin expression in MDA-MB-468 cell：


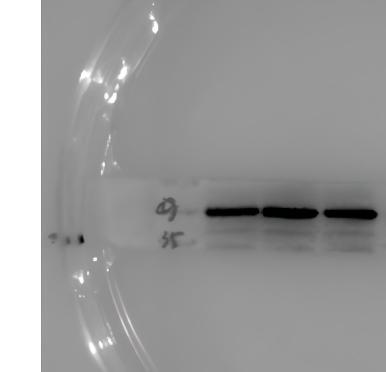


CCAR2 expression in BT549 cell：


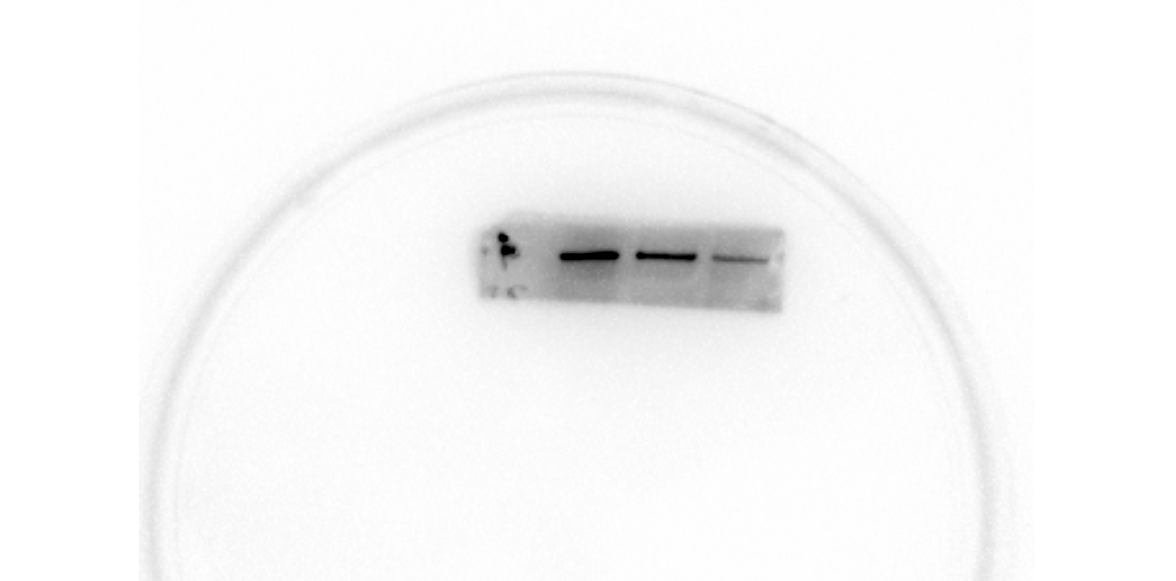


β-actin expression in BT549 cell:


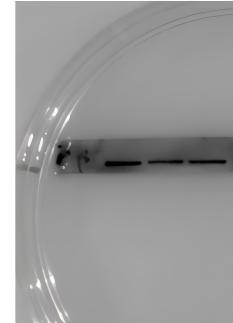


**Figure 7F**

β-actin expression in MDA-MB-468 cell:

**
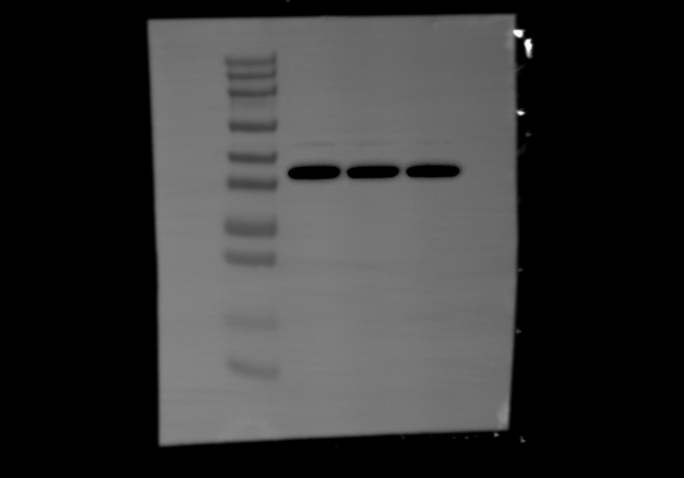
**

β-actin expression in BT549 cell:

**
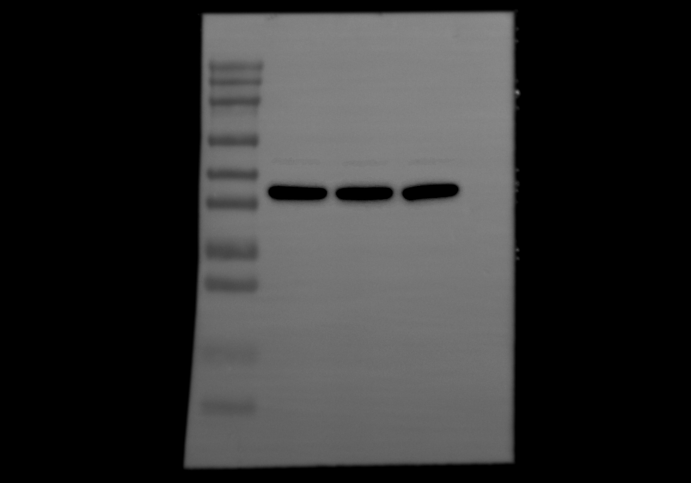
**

Bax expression in MDA-MB-468 cell:

**
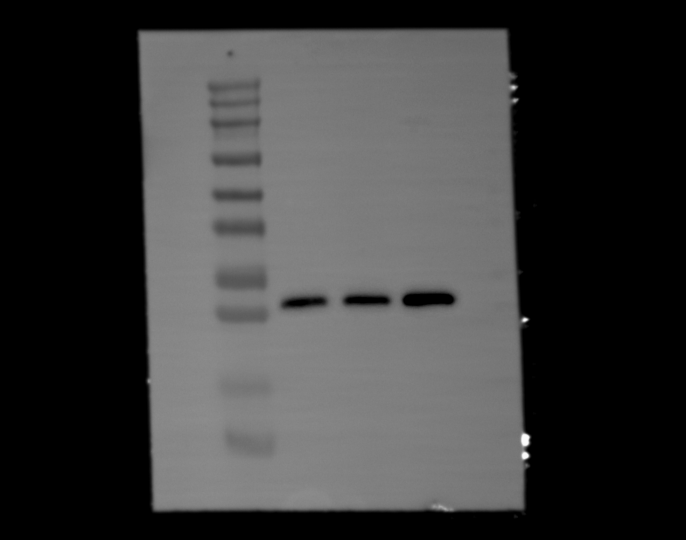
**

Bax expression in BT549 cell:

**
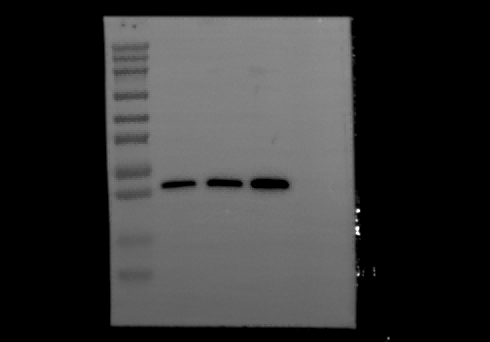
**

Bcl-2 expression in MDA-MB-468 cell:

**
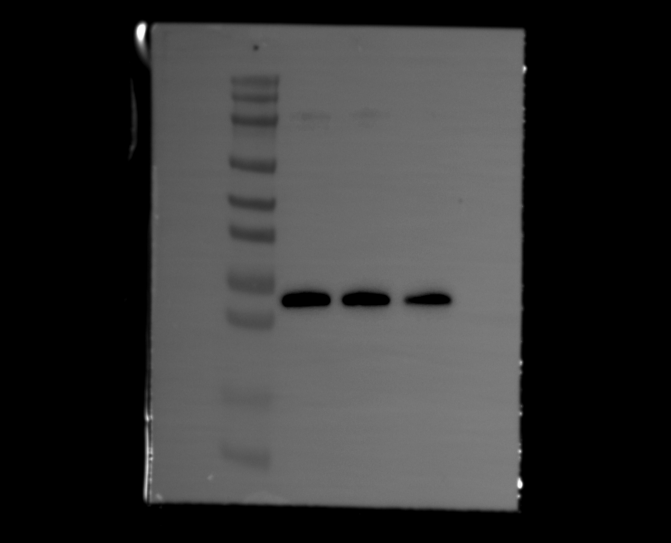
**

Bcl-2 expression in BT549 cell:

**
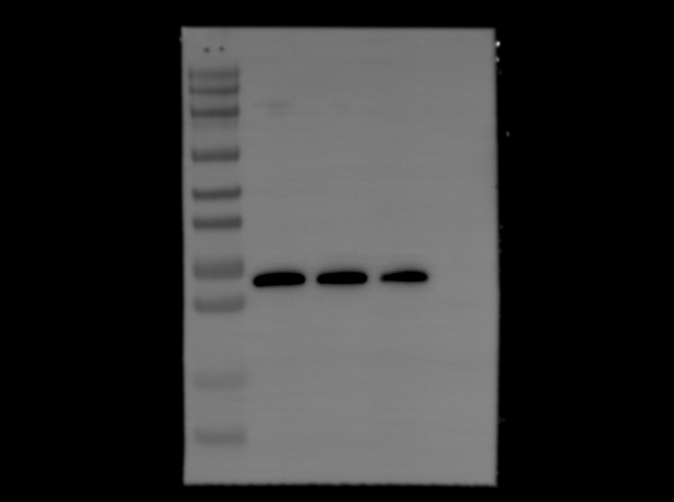
**
