# Supplementary material for: An anoikis-related gene signature predicts prognosis and immunotherapy response, and identifies CCAR2 as a therapeutic target in triple-negative breast cancer
Source: Front Immunol. 2026 May 20;17:1808490. doi: 10.3389/fimmu.2026.1808490 (PMC13229996; doi:10.3389/fimmu.2026.1808490)
Supplement: Supplementary file 2 [file DataSheet2.docx]

Supplementary Material

# Supplementary materials and methods

**1.1 Data sources and processing**

The mRNA sequencing data of TNBC samples were obtained from The Cancer Genome Atlas (TCGA) database ( <https://portal.gdc.cancer.gov> ). The Gene Expression Omnibus (GEO) database was used as the source of the GSE58812 and GSE180286 gene expression datasets. Data from 107 TNBC patients from GSE58812 and two TNBC patients from GSE180286 were extracted for analysis. All gene expression data were annotated using the GPL570 platform.

**1.2 Association analysis of the** **risk signature with clinical characteristics**

We assessed the correlation between the prognostic risk signature and key clinicopathological characteristics and further stratified patients according to various clinical parameters. Using the Wilcoxon rank-sum test, we compared the distribution of risk scores across subgroups and visualized the results as box plots.

**1.3 Analysis of immune cell infiltration and drug Sensitivity**

CIBERSORT analysis, a widely used algorithm for evaluating immune cell infiltration, was employed to quantify the relative abundance of 22 immune cell types. The GDSC database ( <https://www.cancerrxgene.org/> ) was used to analyze drug sensitivity and serve as a comprehensive resource for genomic and drug response data in cancer cell lines. The “pRRophetic” package was used to predict the drug sensitivity of each tumor sample. Potential batch effects were mitigated using the “combat” R package, and the half-maximal inhibitory concentration (IC50) of the drugs was estimated.

**1.4 Gene set variation and enrichment analysis**

Gene Set Variation Analysis (GSVA) and Gene Set Enrichment Analysis (GSEA) are complementary methods for evaluating the enrichment in transcriptome data. Gene sets were obtained from the Molecular Signatures Database (MSigDB; version 7.0). GSVA was used to assess the underlying biological functions, whereas GSEA was employed to delineate signaling pathway disparities between predefined high- and low-risk groups. Pathway enrichment analysis was performed and the results were visualized using the “ggplot2” R package.

**1.5 TMB, MSI, and mutation landscape**

Tumor Mutational Burden (TMB) and Microsatellite Instability (MSI) were calculated to investigate their potential as biomarkers for immunotherapy. TMB was defined as the total number of somatic mutations per megabase, calculated by dividing the number of nonsynonymous mutation sites in a tumor sample by the total length of the sequenced protein-coding region (in megabases) ([1](#_ENREF_1)). The MSI status of the cohort was obtained from a previous study ([2](#_ENREF_2)).

**1.6 Genome‑wide association study analysis**

Potential pathogenic genes were identified using genome-wide association study (GWAS) data, which implicated specific genomic loci in the risk of disease. Based on these loci, CCAR2 and ITGA5 were prioritized as candidate genes for further investigation. Their phenotypic associations were explored by analyzing the Gene Atlas database ( <http://geneatlas.roslin.ed.ac.uk/> ), a resource that quantifies links between millions of genetic variants and hundreds of traits, including 778 phenotypes, using genetic data from 452,264 individuals in the UK Biobank ([3](#_ENREF_3)).

**1.7 Single-cell analysis**

The GSE180286 single-cell dataset was processed using the Seurat pipeline. The gene expression values were log-normalized. Cells were grouped for further analysis, and cellular subpopulations were identified using t-distributed Stochastic Neighbor Embedding (t-SNE). Cell types within each cluster were annotated using the “celldex” package, focusing on those critically involved in the tumorigenesis. Subsequently, marker genes for each cellular subtype were identified from the single-cell expression profiles by applying the Find All Markers function, with the logfc.threshold parameter set to one.

**1.8 Characterization of CCAR2 expression through bioinformatic analysis**

The Human Protein Atlas (HPA) database ( <https://www.proteinatlas.org/> ) provides detailed spatial expression information for over 24,000 human proteins across various tissues and cells. We queried the HPA to analyze CCAR2 expression in the breast cancer dataset. Additionally, the correlation between CCAR2 expression and the stemness index (mRNAsi) in TNBC was assessed using ASSISTANT for Clinical Bioinformatics (<https://www.aclbi.com>), which employs the OCLR algorithm developed by Malta et al ([4](#_ENREF_4)).

**1.9 Cell culture**

Human TNBC cell lines MDA-MB-468, MDA-MB-231, BT549, and HS578T were purchased from Wuhan Servicebio Technology Co. Ltd. MCF-10A cells were obtained from Zhejiang Nuo Bo Biotechnology Co., Ltd. MDA-MB-468, MDA-MB-231, and HS578T cells were cultured in Dulbecco’s modified DMEM medium (DMEM; Gibco) supplemented with 10% fetal bovine serum (Gibco). BT549 cells were maintained in a specific culture medium (Servicebio), and MCF-10A cells were grown in a dedicated medium (Procell). All cell lines were incubated at 37 °C in a humidified atmosphere containing 5% CO₂.

**1.10 cDNA synthesis and Quantitative Real-time PCR**

Total RNA was extracted using the RNeasy Rapid Extraction Kit (GOONIE, China). Following cDNA synthesis using the FastKing RT Kit (Tiangen Biochemical Technology, China), the relative expression of CCAR2 mRNA was quantified using the Talent qPCR PreMix Kit (SYBR Green) with a CFX96 PCR system (Bio-Rad, USA). The thermal cycling conditions were as follows: 95 °C for 15 min, followed by 40 cycles of 95 °C for 10 s and 60 °C for 30 s. Relative mRNA expression level was calculated using the 2^−ΔΔCt^ method. The primer sequences used were: GAPDH: forward 5’-TGCACCACCAACTGCTTAGC-3’, reverse 5’-GGCATGGACTGTGGTCATGAG-3’, CCAR2: forward 5’-AAGGGAGACGCCAGAGCAT-3’, reverse 5’-CATCCAGGGAAGGAGACCAT-3’.

**1.11 Western blot**

Total cellular proteins were extracted using RIPA buffer supplemented with protease inhibitors (Cwbiotech). Protein lysates were mixed with SDS loading buffer (Cwbio) and denatured by heating at 100 °C. After separation on an 8% SDS-polyacrylamide gel (CWBio), proteins were transferred onto a PVDF membrane (Millipore). The membrane was blocked with 5% skim milk for 2 h at room temperature and then incubated overnight at 4 °C with the following primary antibodies: anti-CCAR2 (1:1000, ab215852, Abcam), anti-Bcl-2 (1:1000, ab241548, Abcam), anti-Bax (1:1000, ab182733, Abcam), and anti-β-actin (1:1000, GB15003, Servicebio). After washing, the membranes were incubated with horseradish peroxidase-linked secondary antibodies (1:5,000; Servicebio, GB23303, and GB23302) for 1 h at room temperature. Protein bands were visualized using an ECL chemiluminescence kit (Servicebio) and detected using a ChemiDoc MP Imaging System (Bio-Rad).

**1.12 Calcein-AM/Ethidium Homodimer-I (EthD-I) staining assay**

After detachment or reattachment, the cells were stained by adding 1μL of Calcein-AM and 1μL of Ethidium Homodimer-1 (EthD-1) to the culture medium. Staining was performed in the dark, followed by a 60-minute incubation at 37 °C in a CO2 incubator. Stained cells were visualized and imaged using a fluorescence microscope.

**1.13 Measurement of caspase-3 activity and mitochondrial membrane potential**

Cells were co-stained with MitoTracker Deep Red 633, a mitochondrial membrane potential-sensitive far-red fluorescent probe, and GreenNuc™ Caspase-3 Substrate, a fluorogenic substrate for Caspase-3 enzyme, to assess mitochondrial membrane potential and Caspase-3 activity in apoptotic cells, respectively. Following incubation according to the manufacturer's protocol (C1073S, Beyotime Biotechnology Co., Ltd. Shanghai, China), the cells were smeared onto slides, visualized, and imaged under a fluorescence microscope.

**1.14 Enzyme-linked immunosorbent assay (ELISA)**

Following the addition of reagents to the standard and sample wells, 100 µL of detection antibody was added to all wells except the blank wells. The plates were then incubated at 37 °C for 1 h. We then added substrate solutions A and B, incubated the plate at 37 °C for 15 min, stopped the reaction with a stop solution, and measured the optical density (OD) using a microplate reader.

**1.15 Immunofluorescence (IF) staining**

After permeabilization with 0.1% Triton X-100, the cells were blocked with goat serum and then incubated overnight at 4 °C with the following primary antibodies at the specified dilutions: anti-ALDH1A1 (1:400, CST 36671), anti-Oct4 (1:500, HUABIO ET1612-20), anti-Nanog (1:200, HUABIO ET1610-2), anti-CD24 (1:500, Invitrogen 14-0242-82), and anti-CD44 (1:500, Abcam ab254530). The cells were then incubated for 1 h at room temperature with species-matched fluorescent secondary antibodies, goat anti-mouse IgG (1:1000, Abcam ab150113) or donkey anti-rabbit IgG (1:1000, Abcam ab150075). The fluorescence intensities of ALDH1A1, Oct4, Nanog, and CD44/CD24 were quantified using ImageJ software.

References

1. Jardim DL, Goodman A, de Melo Gagliato D, Kurzrock R. The Challenges of Tumor Mutational Burden as an Immunotherapy Biomarker. Cancer Cell. (2021) 39(2):154–73. doi: 10.1016/j.ccell.2020.10.001.

2. Bonneville R, Krook MA, Kautto EA, Miya J, Wing MR, Chen HZ, et al. Landscape of Microsatellite Instability Across 39 Cancer Types. JCO Precis Oncol. (2017) 2017. doi: 10.1200/po.17.00073.

3. Canela-Xandri O, Rawlik K, Tenesa A. An atlas of genetic associations in UK Biobank. Nat Genet. (2018) 50(11):1593–9. doi: 10.1038/s41588-018-0248-z.

4. Malta TM, Sokolov A, Gentles AJ, Burzykowski T, Poisson L, Weinstein JN, et al. Machine Learning Identifies Stemness Features Associated with Oncogenic Dedifferentiation. Cell. (2018) 173(2):338–54.e15. doi: 10.1016/j.cell.2018.03.034.
