# Supplementary material for: An anoikis-related gene signature predicts prognosis and immunotherapy response, and identifies CCAR2 as a therapeutic target in triple-negative breast cancer
Source: Front Immunol. 2026 May 20;17:1808490. doi: 10.3389/fimmu.2026.1808490 (PMC13229996; doi:10.3389/fimmu.2026.1808490)
Supplement: Supplementary file 3 [file Table1.docx]

Supplementary Material

**Supplementary Table 1. The expression correlation between CCAR2 and 26 immune checkpoint genes in basal-like breast cancer.**

| **Gene symbol** | **Descriptions** | **Rho** | ***P*** |
| --- | --- | --- | --- |
| BTN2A1 | Butyrophilin Subfamily 2 Member A1 | 0.361 | 3.71e-07*** |
| BTN2A2 | Butyrophilin Subfamily 2 Member A2 | 0.230 | 1.47e-03* |
| BTN3A1 | Butyrophilin Subfamily 3 Member A1 | 0.230 | 1.53e-03* |
| CD209 | CD209 Molecule | 0.196 | 6.96e-03* |
| CD274 | CD274 Molecule | 0.148 | 4.33e-02* |
| CD276 | CD276 Molecule | 0.372 | 1.44e-07*** |
| CD40 | CD40 Molecule | 0.296 | 3.78e-05*** |
| CD70 | CD70 Molecule | 0.191 | 8.66e-03** |
| CD80 | CD80 Molecule | 0.185 | 1.09e-02* |
| CD86 | CD86 Molecule | 0.242 | 8.13e-04*** |
| CTLA4 | Cytotoxic T-Lymphocyte Associated Protein 4 | 0.160 | 2.87e-02* |
| HAVCR2 | Hepatitis A Virus Cellular Receptor 2 | 0.234 | 1.24e-03** |
| HLA-C | Major Histocompatibility Complex, Class I, C | 0.181 | 1.31e-02* |
| HLA-DOA | Major Histocompatibility Complex, Class II, DO Alpha | 0.172 | 1.78e-02* |
| HLA-DPB1 | Major Histocompatibility Complex, Class II, DP Beta 1 | 0.157 | 3.11e-02* |
| HLA-DQA1 | Major Histocompatibility Complex, Class II, DQ Alpha 1 | 0.164 | 2.44e-02* |
| HLA-DQB1 | Major Histocompatibility Complex, Class II, DQ Beta 1 | 0.175 | 1.65e-02* |
| HLA-DRB1 | Major Histocompatibility Complex, Class II, DR Beta 1 | 0.143 | 4.98e-02* |
| HLA-DRB5 | Major Histocompatibility Complex, Class II, DR Beta 5 | 0.181 | 1.28e-02* |
| HLA-E | Major Histocompatibility Complex, Class I, E | 0.156 | 3.26e-02* |
| ICOSLG | Inducible T Cell Costimulator Ligand | 0.161 | 2.78e-02* |
| LAG3 | Lymphocyte Activating 3 | 0.212 | 3.49e-03** |
| LGALS9 | Galectin 9 | 0.152 | 3.73e-02* |
| PDCD1 | Programmed Cell Death 1 | 0.172 | 1.84e-02* |
| TNFRSF14 | TNF Receptor Superfamily Member 14 | 0.302 | 2.52e-05*** |
| TNFRSF4 | TNF Receptor Superfamily Member 4 | 0.271 | 1.73e-04*** |

Note: **P*<0.05, ***P*<0.01, ****P*<0.001.
